# Oral manifestations of HIV/AIDS in Asia: Systematic review and future research guidelines

**DOI:** 10.4317/jced.52127

**Published:** 2015-07-01

**Authors:** Gaurav Sharma, Sukhvinder-Singh Oberoi, Puneeta Vohra, Archna Nagpal

**Affiliations:** 1 Reader, Department of Oral Medicine, S.R. Dental College, Faridabad, Haryana, India-121002; 2 Reader, Department of Public Health Dentistry, S.R. Dental College, Faridabad, Haryana, India-121002; 3 Reader, Department of Oral Medicine, S.G.T. Dental College, Gurgaon, Haryana, India; 4 Reader, Department of Oral Medicine, P.D.M. Dental College, Bahadurgarh, Haryana, India

## Abstract

**Objectives:**

The authors have conducted a systematic review of oral manifestations of HIV from studies conducted in Asia to establish the characteristics and prevalence of individual oral manifestations in Asia, and to assess the direction of future research studies on oral manifestations of HIV in Asia.

**Material and Methods:**

The electronic retrieval systems and databases searched for relevant articles were PubMed [MEDLINE], EBSCO, and EMBASE. The search was for limited articles published in English or with an English abstract and articles published during the period January 1995 to August 2014. The authors reached a final overall sample of 39 studies that were conducted in Asia.

**Results:**

The median population size among all studies was 312.7 patients. Oral candidiasis [OC] was the most common oral manifestation [37.7%] in studies conducted in Asia. The overall prevalence of oral hairy leukoplakia and melanotic hyperpigmentation was computed to be 10.1% and 22.8% respectively. Thailand and India are primarily countries with maximum research on oral manifestations.

**Conclusions:**

The research on oral manifestations of HIV in Asia has to upgrade to more interventional and therapeutic studies rather than the contemporary cross- sectional epidemiological descriptive studies. The authors have given suggestions and future directions for the implementation of clinical research of oral manifestations in HIV patients.

** Key words:**Oral manifestations, HIV/AIDS, Asia, Systematic review.

## Introduction

Human Immunodeficiency Virus [HIV] is a massive and byzantine challenge for the public health system. The spread of HIV infection in Asian countries is a major concern that is still showing a rising trend (1). Despite a 26% reduction in HIV infections since 2001, the pandemic still outpaces the response and half of the people [49%] in Asia pacific region are unable to access the anti-retroviral therapy. The overall national prevalence in Asian countries is low, a misleading true statistic, that tends to camouflage the real pandemic threat. The huge volume of Asian population ensures that even a low prevalence transmutes into a colossal HIV infected population in numbers. HIV infection is of portentous significance as majority of the patients belong to economically and reproductively active group within their communities which can have a devastating effect on the socio-economic status of nation.

Oral manifestations in HIV infection have been well documented as early markers of HIV infection and progression (2). Oral manifestations of HIV/AIDS [Acquired immunodeficiency syndrome] are significant as they may affect the patients’ quality of life and can be used to assess the status of immunosuppression and determine the prognosis of the disease (3). Early diagnosis and appropriate treatment of oral lesions have great influence on patients’ general health and can reduce the mortality rate of the disease. Thus it becomes imperative for all health care workers to be equipped with the necessary knowledge and expertise to manage oral manifestations of HIV infection.

There are considerable regional variations in the oral manifestations of HIV infection, depending both on the populations studied and on the clinical heterogeneity (3). The oral manifestations in Asian countries as compared to western countries and other developing nations in Africa and Latin Americas are relatively different (2). The constraint of resources in Asian countries hinders the possibility of providing an effective health care system. Though many studies have been conducted on oral manifestations in HIV, the research in Asia is currently at a pivotal juncture as the existing research is getting repetitive. Currently, Asian studies predominantly focus on prevalence of oral manifestations and assess their association with parameters of HIV infection, sociodemographic data and deleterious habits. An oral lesion index is required that can assist health care workers to identify the oral manifestations and thus evaluate the predictability of the immune status in HIV patients especially in a resource constrained settings. The authors have hereby conducted a systematic review of oral manifestations of studies conducted in Asia to establish the characteristics and prevalence of individual oral manifestations in Asia, and to assess the direction of future research studies on oral manifestations of HIV in Asia. The author’s primary aim is to provide a computed prevalence of the oral manifestations of HIV/AIDS in Asia that would help in the possibility of formulation of oral lesion index in the foreseeable future.

## Material and Methods

The systematic review was based on the ongoing research on oral manifestations of HIV/AIDS. The geographical region of Asia was considered primarily as the maximum number of HIV patients is currently present in Asia. The PubMed interface of MED-LINE was interrogated by MeSH and free-text words. A comprehensive literature search was executed till August 2014. The MeSH was ‘‘oral, lesions’’, “oral manifestations”, HIV”, “AIDS”. The electronic retrieval systems and databases searched for relevant articles were PubMed [MEDLINE], EBSCO, and EMBASE. The search was for limited articles published in English or with an English abstract and articles published during the period January 1995 to August 2014. Each country located within Asia [countries were included according to United Nations website code 142] was individually typed in concurrence with the above mentioned Mesh words. Only oral epidemiological studies related to oral manifestations of HIV were included. All the studies that had followed presumptive criteria of EEC clearinghouse classification [1993] were included. Articles were manually retrieved from National Medical Library, New Delhi. Relevant cross references of the articles were also retrieved manually or electronically.

The shortlisted studies were screened and independently categorized by all the authors. Any disagreement between the authors regarding article and data extraction was sorted. In reading the articles, the reference lists were checked to identify any other articles that may have been relevant to the research question or provided additional information. Individual prevalence rates were observed and were computed manually wherever it was required. The exclusion criteria to ensure uniformity of the studies were:

1. Studies on oral manifestations done outside Asia were excluded. Russia was excluded due to geographical overlap with Europe.

2. Exclusive HIV paediatric studies were excluded.

3. Interventional studies for oral manifestations in HIV patients were not included.

4. Individual case reports were excluded.

The authors reached a final overall sample of 39 studies (4-42) that were conducted in Asia after applying the exclusion criteria (Methodology described in  Table 1).

**Table 1:**
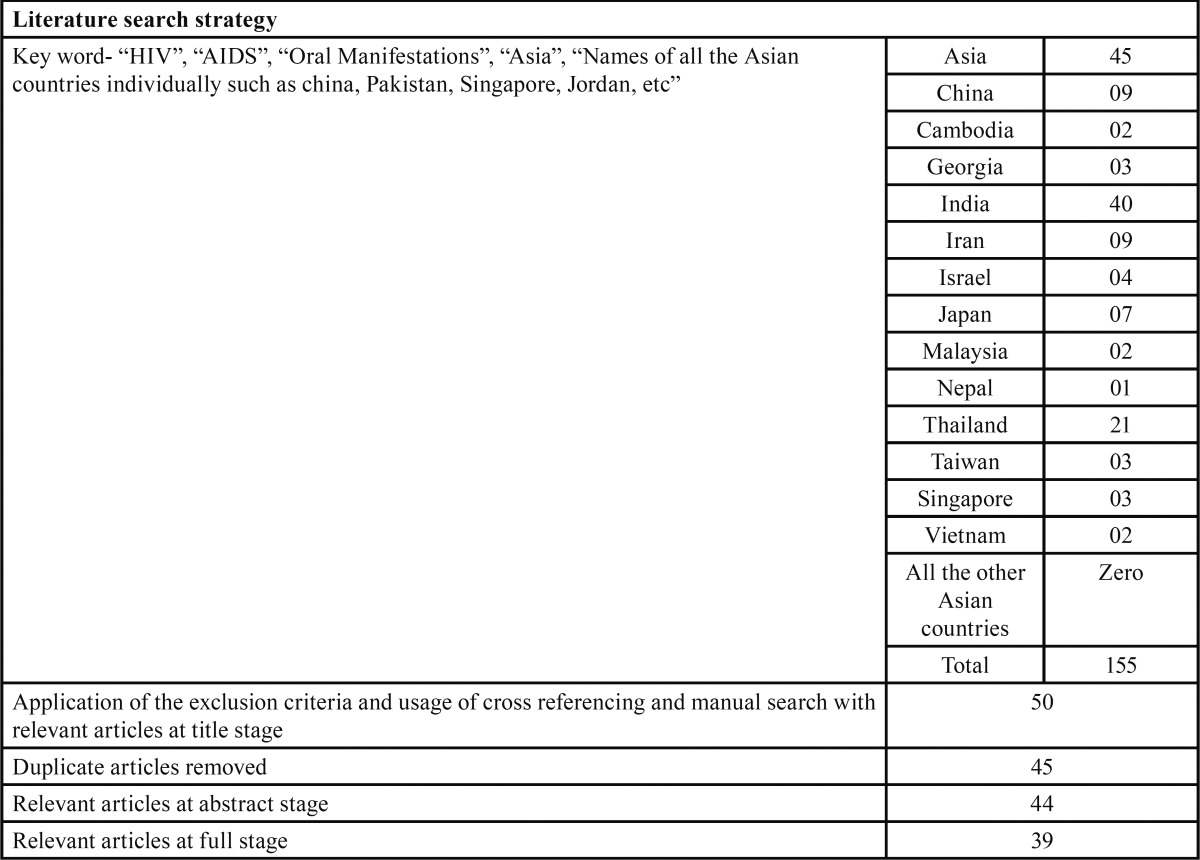
Depicts the key words and their combinations used in the literature search for oral manifestations in HIV.

## Results

Tabulation of the research studies (4-42) conducted on oral manifestations in Asia was done ( Table 2,  Table 2 (Cont)). Majority of the studies [25/39; 64.1%] have been conducted in India and Thailand. No research studies on oral manifestations of Asia were documented in 37 countries. Population of sample study ranged from 32 patients (8) to 3729 patients (38). The median population size among all studies was 312.7 patients. The primary mode of transmission of HIV in studies conducted in Asia is predominantly heterosexual route [overall 76.5%]. Males were more affected with HIV than females in almost all the studies except in a recent study (41) where all the 292 patients were HIV infected pregnant women. Anti-retro viral therapy [ART]’s usage was documented in twenty one studies [21/39; 53.8%]. ART was observed in these studies with varying percentages and the combined prevalence of patients on ART in Asian studies was observed to be only 35.7%.

**Table 2:**
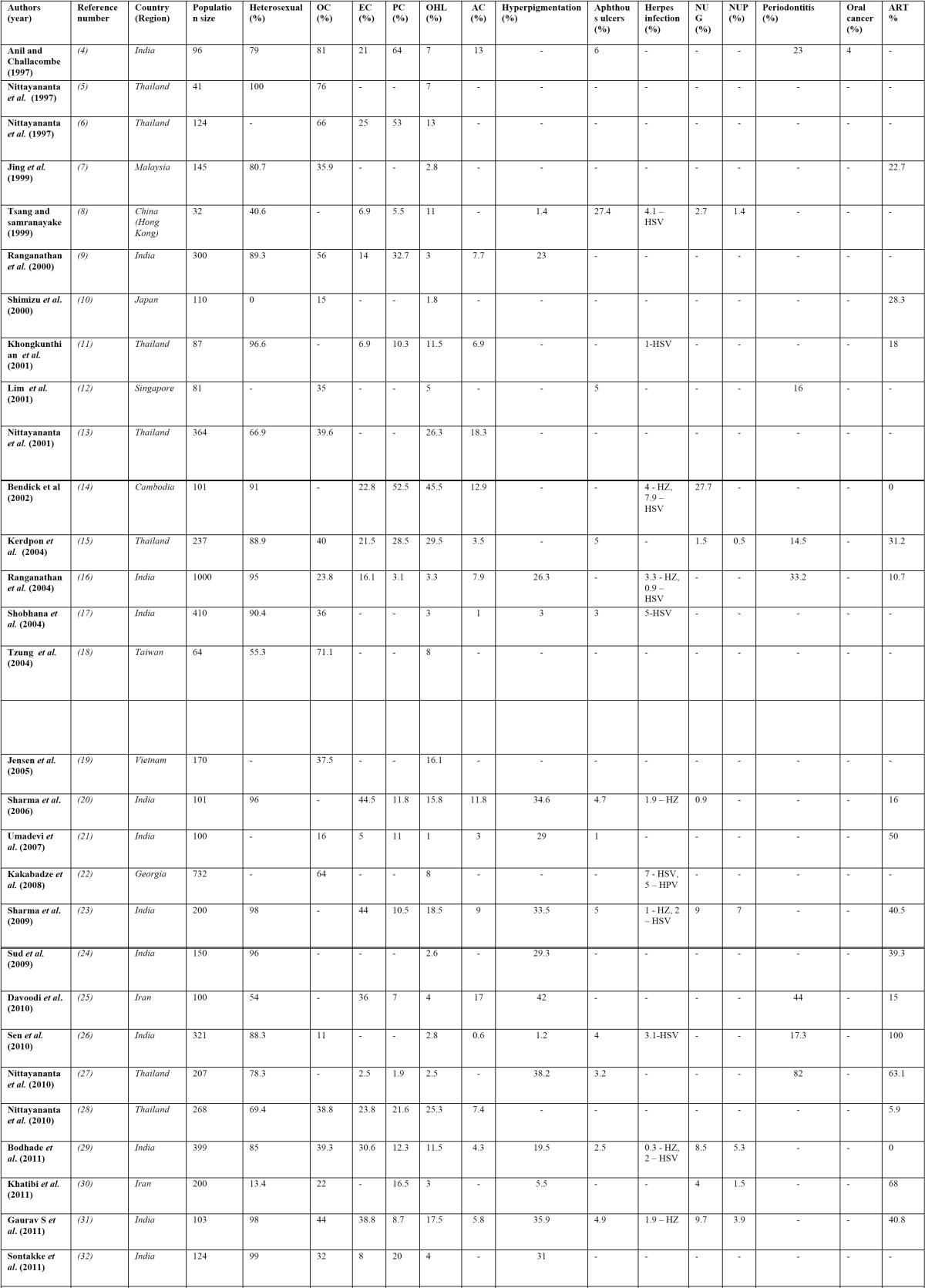
Published studies conducted on oral manifestations in HIV/AIDS included in the review.

**Table 2 (Cont):**
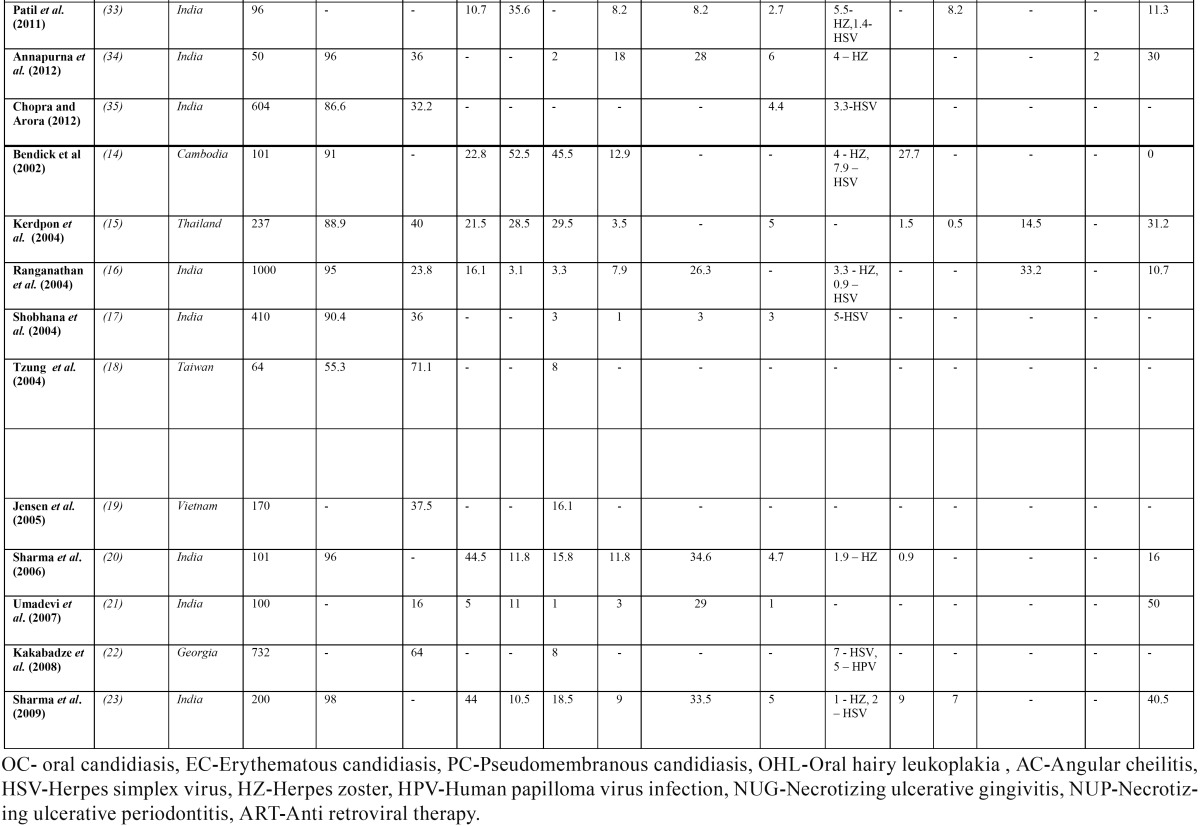
Published studies conducted on oral manifestations in HIV/AIDS included in the review.

-Oral candidiasis

**Figure 1:**
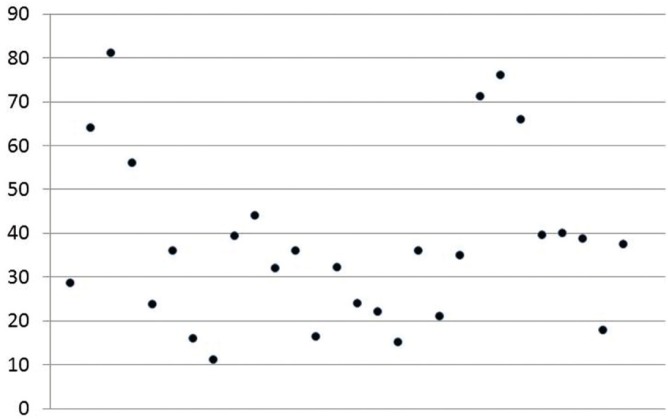
Scatter diagram showing oral candidiasis prevalence in the conducted studies on oral manifestations in HIV.

Oral candidiasis [OC] was the most common oral manifestation [37.7%] in studies conducted in Asia. The exact prevalence was difficult to ascertain in review as some researchers had listed only the subtypes of oral candidiasis. The range of OC varies broad-ly from a high prevalence of 81% (4) to 11% (26) (Fig. 1). Pseudomembranous candidiasis [average prevalence- 20.4%; range 1.9%-64%] and erythematous candidiasis [average prevalence- 20.9%; range 2.5%-44.5%] were equally observed where the subtype was clearly mentioned in the study. Hyperplastic candidiasis was reported infrequently with a low prevalence of 1% from studies conducted by Ranganathan *et al.* (9,16). Angular cheilitis was observed in Asia with an overall prevalence of 8.1%, though Nittayananta *et al.* (13) and Annapurna *et al.* (34) had observed higher prevalence rates of 18.3% and 18% respectively.

-Oral Hairy Leukoplakia 

**Figure 2:**
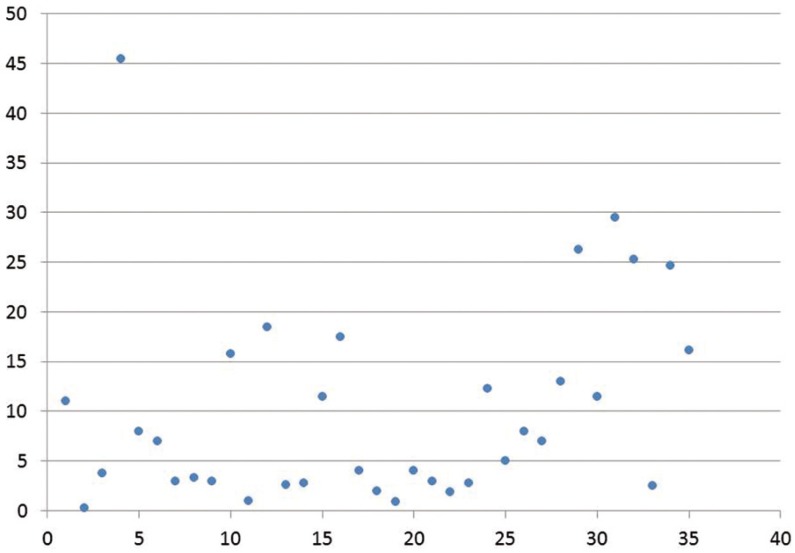
Scatter diagram showing oral hairy leukoplakia prevalence in conducted studies on oral manifestations in HIV.

Oral Hairy Leukoplakia [OHL], caused by Epstein Barr virus [EBV], was the most common oral manifestation in only one study conducted by Khongkunthian *et al.* (11) in Thailand. The combined prevalence of all the studies conducted revealed a value of 10.1% with a range of 0.2% to 45.5% (Fig. 2). Only four studies did not document OHL (33,35,39,42). The prevalence of OHL was higher in few studies varying from 26.3% by Nittayananta *et al.* (13) in 2001 to 45.5% by Bendick *et al.* (14).

-Melanotic hyperpigmentation

The overall prevalence of melanotic hyperpigmentation was computed to be 22.8 % and was observed in twenty studies. Melanotic hyperpigmentation was the most common HIV-associated oral manifestation in studies conducted by Sud *et al.* (24) in 2009, Davoodi *et al.* (25) in 2010 and Nittayananta *et al.* (27) in Thailand. The prevalence of melanotic hyperpigmentation has been higher in studies reported by Sharma *et al.* (20) [34.6%] in 2006 and Nittayananta *et al.* (27) [38.2%] in 2010. However, a low prevalence of 1.4% in Hong Kong documented by Tsang and Samaranayake (8) can be attributed to lack of anti-retroviral therapy in their study sample.

-Oral malignancies

Oral cancer was observed only in two studies with prevalence rates of 4% and 2% (4,34). Anil and Challacombe (4) revealed a high incidence of squamous cell carcinoma in the studied population. Recently, Khatibi *et al.* (30) and Perera *et al.* (37) had reported a prevalence of 1.9% and 1% of oral Kaposi’s sarcoma respectively. The prevalence of Non-Hodgkin’s Lymphoma [NHL] in Asian studies has been found to be very low as compared to developed countries (15). NHL was however observed with a prevalence of 4% in a study conducted by Nittayananta *et al.* (6) in 1997.

-HIV associated periodontal disease

HIV associated periodontal disease includes Necrotizing ulcerative gingivitis [NUG], necrotizing ulcerative periodontitis [NUP] and linear gingival erythema [LGE]. The average prevalence of NUG and NUP have been found to be 7.6% and 4.2% respectively. NUG has been found in a prevalence of 27.7% in Cambodia (14) and 9% in India (23). The prevalence of NUP has typically remained low in studies from Asia with a prevalence of 0.5% [Thailand] (15) 1.5% [Iran] (30), and 3.9% [India] (31). The prevalence of LGE has remained variable with Khatibi *et al.* (30) observing a high prevalence of 22% in their study from Iran, whereas Sontakke *et al.* (32) had documented a low prevalence of 2.4% in their study.

-HIV Salivary gland disease

The prevalence of parotid gland enlargement has typically remained low [around 1%] (15). HIV infection has also been known to cause xerostomia and salivary gland hypofunction. Nittayananta *et al.* (27) had found a significant reduction of both stimulated and unstimulated salivary flow in patients with HIV. Sharma *et al.* (20) had observed a prevalence of around 30% in their cross sectional study and Davoodi *et al.* (25) had documented 20% prevalence of xerostomia.

-Ulcers

The computed prevalence of apthous stomatitis was observed to be 4.1%. However, Tsang and Samaranayake (8) had observed a very high prevalence of 27.4% in their cohort of 32 HIV patients from Hong Kong. Ulceration [Not otherwise specified (NOS)] has also been observed in a prevalence of around 3% (15). However, Nittayananta *et al.* (6) had documented 11% prevalence of ulceration [NOS] in their cohort from Thailand.

-Viral infections

Viral infections [other than EBV and HPV] afflicting the oral mucosa in HIV patients encompasses Herpes simplex virus infec-tions [HSV], Varicella zoster virus[VZV] and cytomegalo virus [CMV]. The prevalence of herpes simplex associated oral ulcers [HSV], Herpes zoster [VZV] and CMV associated salivary gland disease is extremely low in Asian countries. Herpes zoster prevalence in Indian studies ranged from 0.3% to 1.9% (29,31). A Cambodian study had observed a high prevalence of 7.9% HSV infection (14) whereas an Indian study (9) had observed a very low prevalence of 0.9% HSV infection.

-Miscellaneous oral manifestations 

No oral warts were observed or reported from studies in Asian countries (31). Histoplasmosis and penicilliosis caused by Penicillium marneffei were observed in studies conducted in Thailand and were found to be associated with severe immunosuppression (13,15,28). However, reports of penicilliosis were not documented in other Asian studies. Oral submucous fibrosis and homoge-nous leukoplakia were more frequently found in studies conducted in south India by Ranganathan *et al.* (16). Reports of exfoliative cheilitis were scarce in Asian studies (11). Bodhade *et al.* (29) had however observed nine cases of mollascum contagiosum in their study from India.

## Discussion

The systematic review on oral manifestations in HIV revealed a predominant heterosexual transmission mode [76.5%]. However in studies conducted by Tsang and Samaranayake (8) and Bakhshaee *et al.* (42) primary mode of transmission was homosexual route and intravenous drug users. Nittayananta *et al.* (28) in a study in 2010 had compared the oral manifestations with the route of transmission and had observed OC and OHL to be significantly associated with heterosexual route of transmission. Thailand and India are primarily countries with maximum research on oral manifestations. This could possibly be attributed to a larger HIV infected population in these countries. The authors have summarized the trends of oral manifestations of HIV infection in Asia and have proposed guidelines on course of future studies to be conducted in Asia.

The usage of antiretroviral therapy [ART] in this review [35.7%] was also a reflection of the reduced ART usage in Asian countries. The number of patients on ART was extremely variable; it was very difficult to extrapolate the prevalence rates of individual oral manifestations with ART in the review. There was a decrease in oral manifestations when ART usage was more than 50% in study sample. Immune reconstitution disease [IRD], a functionally partial reorganization of immune system, has been observed in individuals on HAART. Salivary gland enlargement, xerostomia, and oral warts also have been suggested to be consequences of IRD (43). Human papilloma virus [HPV] has been more commonly isolated from the oral cavity of HIV positive patients as compared to immunocompetent patients. Umadevi *et al.* (21) had conducted a longitudinal study in south Indian HIV patients on ART and no oral wart was documented in their study. Homosexual men typically have a prevalence of detectable HPV DNA ranging roughly from 80% to 93% (38). In Asian studies predominantly heterosexual population studies have been documented and the possibility of reduced frequency of orogenital contact [as documented in most of studies], could possibly explain the absence of oral warts (21). Studies need to be conducted measuring the stimulated and unstimulated salivary flow in a longitudinal cohort of HIV positive patients initially before HAART has begun and later at six months interval to accurately measure the impact of HAART on xerostomia. No reports of CMV sialadenitis were observed that could possibly be attributed to lack of resources to isolate the virus.

The most common oral manifestation expectedly was OC [with no predominant subtype prevalence]. The difference in prevalence range of OC can be due to differences in the prescribed medications, the stages of the disease or the route of transmission of the infection, and the longevity of HIV infection. Melanotic hyperpigmentation was found with a greater frequency [22.8%] as compared to OHL [10.1%]. This can be attributed to a relatively greater usage of ART that tends to suppress OHL and promotes melanosis (27).

An important aspect of this review was the absence of cases of Kaposi’s sarcoma in Asia, the malignant neoplasia most frequently associated with HIV /AIDS patients. The prevalence of Kaposi’s sarcoma has been found to be higher in Africa and developed world. Shiboski *et al.* (44) had recently postulated that, though oral squamous cell carcinoma [SQCC] has not yet been shown to be associated with HIV ⁄ AIDS in studies, in the era of HAART, patients with HIV disease would live longer and would be more likely to have carcinoma. Factors like greater availability of HAART, high prevalence of oral HPV infection and association of higher usage of tobacco either in smokeless or smoking may combine for a greater risk of developing oral SQCC in Asian countries (45). A recent review of malignancies of Head and neck region revealed 14 cases of oral SQCC outside Asia and also concluded that the HIV-associated carcinoma would occur at a relatively younger age group (45). NHL is recognized as an AIDS defining condition in HIV-infected individuals and is included among the oral lesions associated with HIV infection. The prevalence of NHL was found to be rare in Asia. Nevertheless, the authors postulate that oral malignancies, especially tobacco related, are likely to be documented more frequently in Asia as due to greater usage of ART in future there would be a likelihood of longer life span for HIV patients and thus these immunocompromised patients would be more exposed to initiating oncogenic factors.

Asia accounts for one in six HIV-positive tuberculosis [TB] cases worldwide. Seven Asian countries are present among the world’s 41 high HIV/TB burden countries: Cambodia, China, India, Indonesia, Myanmar, Thailand and Vietnam. Interestingly the prevalence of oral TB lesions in all the studies was extremely rare. There could be a possibility of misdiagnosis of TB ulcer as ulcer of NOS which may be attributed to lack of resources in Asian countries. An early diagnosis and hence early management can help in the better quality of life to these patients.

High rates of failure to follow-up the HIV patients discourage the researchers to conduct longitudinal studies in Asian countries. The main constraint for future longitudinal studies is lack of resources. A concerted effort by the health care workers in conjunction with the government is required to improve antiretroviral therapy allocation to patients to ensure an early management of HIV. According to recent UNAIDS report there is currently a deficit of 3.2 billion $ for the funding for achievement of 2015 tar-get. The only way to breach trajectory of HIV is through increased domestic funding. This can occur only if a greater and consis-tent awareness about the HIV and its lethal complications are done in social media, print and electronic advertisement. Eradication of HIV can be done only through a multifaceted coordinated system where every speciality of health care should be involved actively and make things transpire instead of anticipating for HIV to get subjugated by a HIV vaccine cure or the government of respective countries accomplishing radical procedures.

There were 350000 new HIV infections in Asia and more than 90% of the new HIV infections are accounted by twelve countries in Asia-pacific region namely China, India, Thailand, Pakistan, Indonesia, Cambodia, Malaysia, Nepal, Myanmar, Philippines, Vietnam and Papua New Guinea. New HIV infections are concentrated among people who inject drugs, men who have sex with men and transgender people. Greater efforts should be done to safeguard HIV-related human rights, for people living with HIV and to ensure better availability to anti- HIV medications. A better access to oral health care is of utmost importance to these patients as there in neither cure nor a vaccine yet for HIV patients. However, long term progress would occur only if HIV features prominently in countries’ future development agendas.

The biggest hindrance in providing adequate dental and medical health care other than the financial reasons is the societal stigma associated with HIV. In Indonesia, Malaysia, India and other Asian countries homosexual men face a twofold burden of HIV infection as well as homosexuality, thus constraining the HIV patient totally inaccessible and victimized. Therefore a necessity for a strong establishment or group that concentrates on management and counselling of homosexual HIV positive patient exclusively in Asian countries is required. The elimination of stigma, discrimination and injustice against people affected by HIV in Asian countries though remains a perennial challenging task. A simple inexpensive dental health care with the medical set-up should also be integrated to cater to this unique group of the patients. Efforts should be made to ensure that HIV infected patient’s oral cavity is screened at every regular medical check-up visit. This requires amalgamation of partnership between medical personnel monitoring the HIV infection and dentist (46).

The systematic review conducted on oral manifestations do has some limitations. The computed prevalence of individual oral manifestations is not an exact indication as the clinical heterogeneity in race, habits, level of immunosuppression and gender prevalence in each study can make the comparison of studies very difficult. The ethnic diversities among the Asian population can create a variance of clinical presentation and there is a possibility of inter-examiner variability also. Moreover the different medications of ART do have some variations on the immunity thus influencing the oral cavity. The absolute CD4 counts were not considered by the authors as the data was not universal and standardized in respective countries. The effect of the systemic medication also plays a role in the variations of the oral manifestations.

The formulation of an oral lesion index in the future would be an exciting and major breakthrough in the field of oral epidemiolo-gy in HIV/AIDS (31,47). However for the oral lesion index a combined prevalence of the oral manifestations in a given region is required and thus authors have conducted the review for the initial progress for the formulation of an oral lesion index. The authors suggest that the data derived in this review can subsequently be utilized in ensuing research studies further to create a record for further help in analysis of oral manifestations and the predictability of immune status from oral manifestations.

The research on oral manifestations in Asia has to upgrade to more interventional and therapeutic studies rather than the contemporary cross- sectional epidemiological descriptive studies. The only predicament for ensuing stage of research is “If not now, then when”? The authors have given suggestions and future directions for the implementation of clinical research of oral manifestations in HIV patients.

1. The quantity of conducted studies on oral manifestations from Asia is sufficient but there is unequal distribution with most of studies in India and Thailand. Moreover, the data on oral manifestations in HIV/AIDS infected population is limited from Middle East countries, China and Japan. Research studies need to be conducted in these regions.

2. A formulation of oral lesion index was suggested to predict HIV in resource poor countries with no access to CD4 count or viral load. Correlations between oral manifestations in HIV and absolute CD4 counts have been conducted in Asian countries but there is no clarity in data collection [eg. time duration, frequency, monotherapy or multidrug therapy and uniformity]. However rigorous research methodologies with emphasis on HIV Viral load and CD4/CD8 ratio need to be done. So far, only one study in Asia has correlated oral manifestations of HIV with viral load (31).

3. The guidelines by Shiboski *et al.* (44) in 2009 should be adopted in future studies as compared to EEC clearinghouse [1993].

4. A centralized research authority needs to be instituted in each subregion of Asia [like south Asia, Middle East Asia, Southeast Asia etc.] for conducting research especially on clinical trials for management or oral lesions and thus making it more organized and treatment-centric. This would serve a dual purpose of saving resources as well as prevention of duplication of same study.

5. Studies need to be longitudinal and multicentric to exactly ascertain the prevalence of individual oral manifestations. Focus should also be on microbiological aspects of the studies like detection of strains of candida species as they are being conducted in developed nations (43). However, recently few studies in Asia have measured the stimulated and unstimulated salivary flow (48).

6. Two examiners should preferably do the intraoral examination with blinding of HIV status to exactly ascertain the prevalence of individual oral manifestation as a single examiner might be influenced by HIV status and there is always a risk of bias for over diagnosis of HIV related lesions especially OHL.
